# Association of genetic loci for migraine susceptibility in the she people of China

**DOI:** 10.1186/s10194-015-0553-1

**Published:** 2015-08-01

**Authors:** Qi-Fang Lin, Xian-Guo Fu, Long-Teng Yao, Jing Yang, Luo-Yuan Cao, Yong-Tong Xin, Jun-Xia Hou, Lin-Feng Ye, Gen-Bin Huang

**Affiliations:** Department of Internal Neurology, Ningde Municipal Hospital, Affilliated Hospital of Fujian Medical University, Jiaocheng District, Ningde City, Fujian 352100 China; Laboratory of Cell and Molecular Biology, Ningde Municipal Hospital, Affilliated Hospital of Fujian Medical University, Ningde, Fujian China

**Keywords:** Migraine, Single-nucleotide polymorphism, Susceptibility, She people, rs13208321, rs13208321

## Abstract

**Background:**

The purpose of this study was to investigate the association of the genotype and allele frequencies of the polymorphisms rs4379368, rs10504861, rs10915437, rs12134493 and rs13208321 in She people of China with migraine headache susceptibility. The five alleles were previously identified as being associated with migraine in a Western population, but it was not known if this association would hold in a She population. rs4379368 is in the succinic HMG coenzyme A transferase (*C7orf10*) gene; rs10504861 is near the matrix metallopeptidase 16 (*MMP16*) gene; rs10915437 is near the adherens junctions associated protein 1 (*AJAP1*) gene; rs12134493 is upstream of the tetraspanin 2 (*TSPAN2*) gene; and rs13208321 is within the four and a half LIM domains protein 5 (*FHL5*) gene.

**Methods:**

This was a case-controlled study conducted in She people of Fujian province in China. Polymerase chain reaction-restriction fragment length polymorphism and direct sequencing were performed. Univariate and multivariate analyses were used to assess the association of the different genotypes of each SNP with migraine.

**Results:**

The rs4379368 T allele was not in Hardy-Weinberg equilibrium and was more common than the C allele in subjects with migraine (58.7 %; *P* = 0.049), possibly suggesting a selection bias for T allele in this population. In support of this, the CT and TT genotypes were more frequent in the migraine compared with the control groups (54.0 % and 31.7 % vs. 48.0 % and 28.7 %, respectively; *P* = 0.019). These genotypes were also more common in females with migraines than females without migraines (53.8 % and 30.9 % vs. 46.7 % and 27.6 %; *P* = 0.026). Univariate and multivariate analyses found the CC genotype of rs4379368 and AA or AG genotype of rs13208321 were associated with a reduced risk of migraine (*P* values ≤0.039).

**Conclusions:**

Our findings suggest that rs4379368 and rs13208321 are potential genetic markers for migraine in this She population. The findings of this study and others indicate important differences between ethnic populations in regard to genetic markers of migraine susceptibility.

**Electronic supplementary material:**

The online version of this article (doi:10.1186/s10194-015-0553-1) contains supplementary material, which is available to authorized users.

## Background

Migraine headache refers to a common, chronic, and recurrent headache that significantly impacts the ability to work, results in greater health care resource utilization and cost, and a reduced quality of life [1, 2]. A survey study on migraine, conducted in USA in 2010, showed the prevalence of migraine was 13.2 % in the total population, 8.6 % in males, and 17.5 % in females [3].

The etiology and pathogenesis of migraine are still poorly understood [4]. Epidemiological studies found that there is a genetic and familial predisposition for migraine [5–7]. We previously performed an epidemiological study in the She ethnic minority population from Fujian province in China [5]. She people have their own special lifestyle, cultural heritage, and genetic background, which are different from those of Han people and other Asians. The study was a cross-sectional survey in which we evaluated the prevalence of migraine within the She population. We found the prevalence of migraine was about 10.5 %.

Recently, common genetic variants were identified that are associated with migraine susceptibility through twin, family clustering, and genome wide association studies of patients with migraine (with and without aura) [8, 9]. Several genome-wide association studies have investigated risk variants associated with migraines [10, 11]. Recently, Anttila et al. [11] conducted a large migraine meta-analysis that included 29 studies and 23285 patients with migraines and 95425 controls. They identified 12 single nucleotide polymorphisms (SNPs) that were associated with migraine susceptibility. Five of these SNPs had never previously been associated with the condition. They are: rs4379368 located in an intron of the succinic HMG coenzyme A transferase (*C7orf10*) gene; rs10504861 located close to the matrix metallopeptidase 16 (*MMP16*) gene; rs10915437 positioned in the vicinity of the adherens junctions associated protein 1 (*AJAP1*) gene; rs12134493 located upstream of the tetraspanin 2 (*TSPAN2*) gene; and rs13208321 located within the four and a half LIM domains protein 5 (*FHL5*) gene. To date only one study performed in a Spanish population has further tested the association of these five polymorphisms with susceptibility and/or pathogenesis of migraine [12], and no studies have evaluated these polymorphisms in a Chinese population.

The purpose of the current study was to evaluate the association of the newly identified five loci from the study of Anttila et al. with migraine in a She population. We chose these five alleles as there is no information regarding their association with migraine in non-Western ethnic backgrounds, and this information may help to give insight into what polymorphisms may influence migraine independent or dependent upon ethnicity.

## Methods

### Subjects

The study recruited subjects (*N* = 600) from the population of our prior study [5]. Migraine was diagnosed according to the Diagnostic criteria for Migraine developed by the Headache Classification Committee of the International Headache Society in 2004 [13]. Control subjects (*n* = 300) had no personal history or family history of chronic headache. Patients (*n* = 300) and controls were matched in age and gender. This study was approved by the Ethics Committee of our hospital (The IRB of Ningde Municipal Hospital, Affilliated Hospital of Fujian Medical University) and written informed consent was obtained from each patient. The IRB of Ningde Municipal Hospital, Affilliated Hospital of Fujian Medical University.

### Polymorphism analysis

Peripheral venous blood (1–3 mL) was collected and whole blood genomic DNA was extracted and stored at −20 °C.

Polymerase chain reaction-restriction fragment length polymorphism (PCR-RFLP) was used to detect the genotypes of rs4379368, rs10504861 and rs10915437, and direct sequencing was used to evaluate rs12134493 and rs13208321. The findings of the PCR-RFLP were confirmed by directly sequencing randomly selected samples, which was performed by Fuzhou Di’en Biotech Co., Ltd. (Fujian, China). The possible genotypes or rs12134493 were possible AA, AC and CC, and for rs13208321 were AA, AG and GG.

The primers used for the polymerase chain reaction (PCR) were designed by our group and synthesized in Shanghai Sangon Biotech Co., Ltd. (Shanghai, China). Table 1 presents the sequence of different primers used for amplification of DNA, PCR-RFLP, and sequencing analysis. PCR analysis used the following conditions: denaturation at 94 °C for 5 min, 30 cycles of denaturation at 94 °C for 30 s, annealing at 58 °C for 45 s and extension at 72 °C for 60 s, and a final extension at 72 °C for 5 min. The reaction mixture for PCR (25 μL) included ddH_2_O (18 μL), 10 × Buffer (2.5 μL), 2.5 mM dNTP (2 μL), 50 μM/μL forward primer (0.5 μL), 50 μM/μL reverse primer (0.5 μL), 5 units/μL Taq polymerase (0.25 μL) and 50 ng/μL DNA template (2 μL). Following amplification, 5 μL of PCR product was analyzed by 1 % agarose gel electrophoresis.

**Table 1:** Primers used to expansion of 5 polymorphic sites by PCR

Polymorphisms	Primers	Sequence
rs4379368	Forward	5′-AGTGGGCTTTCATTCTGGAA-3′
	Reverse	5′-AAGGGCCTGAAATCTAATTCC-3′
rs10504861	Forward	5′-CGTAAGAGTAATATATTGGCCCA-3′
	Reverse	5′-TTCTCAATTTTGTGGTTACATGC-3′
rs10915437	Forward	5′-TCCTTAAGGTTCTGGCTGGGT-3′
	Reverse	5′-TGGCCCTCACTAAGGGTGATT-3′
rs12134493	Forward	5′-TTGTTTTCTGTGCCCGACAT-3′
	Reverse	5′-GAGGAAATAGAAGTGTGGGGA-3′
rs13208321	Forward	5′-CGCCATTTCAGAGCTGTCA-3′
	Reverse	5′-TTTTGGTCATGTCCCTTCCT-3′

Products from PCR for rs4379368, rs10504861 and rs10915437 were subjected to incubation with restriction endonuclease to detect the presence of the different polymorphisms. The reaction mixture (20 μL) included ddH_2_O (7.5 μL), products from PCR (10 μL), Buffer (2 μL), restriction endonuclease (0.5 μL), either HphI or Tail (Thermo Scientific, Waltham, MA, USA) or MspA1I (New England Biolabs, Ipswich, MA, USA). Incubation was done at 37 °C for 8–16 h. Incubation with Tail restriction endonuclease was done for 8–16 h at 65 °C. The digested products were separated by agarose electrophoresis. The specific possible restriction patterns are presented in Additional file 1.

### Statistical analyses

Categorical variables were presented as numbers and percentages, and continuous variables were shown as mean and standard deviation. *T*-test was used to compare age difference between migraine and control groups. Chi-square goodness-of-fit tests were performed to test violation of Hardy-Weinberg equilibrium by comparing observed numbers to expected values of the three genotypes for each polymorphism. Univariate logistic regression was performed to examine the association between the genotypes of the different SNPs and migraine status. Multiple logistic regression analysis was performed by adjusting for age and gender. In addition, the migraine group was further divided into subgroups according to clinical characteristics of migraine. The distributions of genotype frequencies were compared between the genotypes of the SNPs and migraine-related characteristics. Post-hoc power analysis revealed the power was 0.99, 0.89, 0.98 and 0.94 for detecting the statistical significance of associations between migraine status and genotypes of rs4379368, rs10915437, rs12134493 and rs13208321, respectively. All statistical analyses were carried out with IBM SPSS statistical software version 22 for Windows (IBM Corp., New York, USA). Statistical significance was defined as *P* value <0.05.

## Results

### Study subjects

The demographics of the subjects were similar between groups (Table 2). The mean age was about 45 years and the majority of subjects were female (≥70 %).

**Table 2:** Description of sample demographic characteristics

	Migraine (*n* = 300)		Control (*n* = 300)		
	n	%	n	%	*P* value
Gender					0.236
Male	77	25.7	90	30.0	
Female	223	74.3	210	70.0	
Age^a^	46.4	13.0	44.4	13.4	0.064

^a^Shown as mean and standard deviation

### Hardy-Weinberg equilibrium

Not all of the SNPs were found to be in Hardy-Weinberg equilibrium. The genotypes of SNPs rs4379368 (*P* = 0.049) and rs10504861 (*P* = 0.007) in the migraine group were out of Hardy-Weinberg equilibrium (Table 3). The genotypes of rs10504861 were also out of Hardy-Weinberg equilibrium in the control group (*P* = 0.018); consequently, this SNP was excluded from subsequent statistical analyses.

**Table 3:** Allele frequencies and testing for Hardy-Weinberg equilibrium of selected SNP markers

		Migraine		Control	
SNP	Allele^a^	MAF^b^	*P* value	MAF^b^	*P* value
rs4379368	C/T (T)	352 (58.7 %)	0.0494^c^	316 (52.7 %)	0.5187
rs10504861	C/T (C)	535 (89.2 %)	0.0074^c^	538 (89.7 %)	0.0180^c^
rs10915437	A/G (A)	396 (66.0 %)	0.4905	407 (67.8 %)	0.1148
rs12134493	A/C (C)	573 (95.5 %)	0.5980	561 (93.5 %)	0.2286
rs13208321	A/G (G)	584 (97.3 %)	0.6351	569 (94.8 %)	0.1577

^a^Major allele was indicated in parenthesis

^b^ *MAF* major allele frequency; both number of the major allele and percentage in the particular group were shown

^c^Violation of Hardy-Weinberg equilibrium

### Allele frequency in migraine and control groups

The frequency of T allele of rs4379368 was significantly higher in the migraine compared with the control group (58.7 % vs. 52.7 %; *P* = 0.036) (Table 3). The C allele frequency was more frequent in the migraine then the control groups for rs12134493 (95.5 % vs. 93.5 %, respectively; *P* = 0.026). Significant associations between allele frequencies and migraine status were found for rs12134493 among males (*P* = 0.041), and for rs4379368 (*P* = 0.042) and rs13209321 (*P* = 0.026) among females (data not shown).

### Distribution of genotypes of SNPs

A greater percentage of subjects carried the CC genotype of rs4379368 in the control group than those in the migraine group (23.3 % vs. 14.3 %, respectively). Univariate analysis found that participants carrying the CC genotype of rs4379368 were less likely to have migraine (odds ration [OR] = 0.56; 95 % confidence interval [CI], 0.34-0.90; *P* = 0.016) (Table 4). Mutlivariate analysis, which controlled for age and gender, also found rs4379368 was associated with a lower risk of migraine (OR = 0.52; 95 % CI, 0.32-0.85; *P* = 0.008). Univariate and multivariate analyses found that the AA and AG genotypes of rs13208321e were associated with lower odds of migraine (*P* values ≤0.047), but found no increased or decreased risk of migraine with rs4379368 or rs13208321 genotypes.

**Table 4:** Comparison of distribution of genotypes of selected SNP markers between migraine and control group

	All							
	Migraine (*n* = 300)		Control (*n* = 300)		Univariate analysis		Multivariate analysis^a^	
SNP	n	%	n	%	OR (95 % CI)	*P* value	OR^a^ (95 % CI)	*P* value
rs4379368								
CC	43	14.3	70	23.3	0.56 (0.34, 0.90)	0.016	0.52 (0.32, 0.85)	0.008
CT	162	54	144	48	1.02 (0.71, 1.47)	0.923	0.98 (0.68, 1.43)	0.932
TT	95	31.7	86	28.7	1.00		1.00	
rs10915437								
GG	32	10.7	37	12.3	0.97 (0.57, 1.65)	0.919	0.98 (0.58, 1.67)	0.948
AG	140	46.7	119	39.7	1.32 (0.94, 1.86)	0.107	1.35 (0.96, 1.91)	0.086
AA	128	42.7	144	48	1.00		1.00	
rs12134493^b^								
AA or AC	26	8.6	39	13	0.64 (0.38, 1.07)	0.090	0.64 (0.38, 1.08)	0.092
CC	274	91.3	261	87	1.00		1.00	
rs13208321^c^								
AA or AG	16	5.3	29	9.7	0.53 (0.28, 0.99)	0.047	0.51 (0.27, 0.97)	0.039
GG	284	94.7	271	90.3	1.00		1.00	

*OR* odds ratio, *CI* confidence interval

^a^Age and gender were included as covariates

^b^Genotyp AA and AC were combined into one group due to small sample size

^c^Genotype AA and AG were combined into one group due to small sample size

### Comparisons of SNPs between migraine patients with or without aura, and controls

Migraine patients were stratified based on the presence or absence of aura. Fewer people in the migraine without aura group carried the CC genotype of rs4379368 than those in the migraine with aura (22.6 vs. 12.2 %; *P* = 0.011) and control (23.3 vs. 12.2 %; *P* = 0.003) groups (Table 5). Similarly, the C-allele frequency of rs4379368 was lower in the migraine without aura (38.4 %) compared with the migraine with aura (52.4 %) and control (47.3 %) groups (*P* = 0.005). No other SNP was associated with aura, and none of the SNPs were associated with additional features including family history, frequency, duration, laterality, pulsing pain, nausea, photophobia, phonophobia, or physical activity (Additional file 1: Table S1).

**Table 5:** Comparisons of migraine with or without aura, and controls

	Migraine					
	Aura (*n* = 62)		Without aura (*n* = 238)		Control (*n* = 300)	
SNP	n	%	n	%	n	%
rs4379368						
CC	14	22.6	29	12.2^a^	70	23.3^b^
CT	37	59.7	125	52.5	144	48.0
TT	11	17.7	84	35.3	86	28.7
rs10504861						
CC	56	90.3	187	78.6	245	81.7
CT	4	6.5	45	18.9	48	16.0
TT	2	3.2	6	2.5	7	2.3
rs10915437						
AA	26	41.9	102	42.9	144	48.0
AG	30	48.4	110	46.2	119	39.7
GG	6	9.7	26	10.9	37	12.3
rs12134493						
AA	0	0.0	1	0.4	0	0.0
AC	6	9.7	19	8.0	39	13.0
CC	56	90.3	218	91.6	261	87.0
rs13208321						
AA	0	0.0	0	0.0	2	0.7
AG	1	1.6	15	6.3	27	9.0
GG	61	98.4	223	93.7	271	90.3

^a^ *P* < 0.05 for Aura vs. Non-aura

^b^ *P* < 0.05 for Non-aura vs. control

## Discussion

Our study evaluated the association of the newly identified rs4379368, rs10504861, rs10915437, rs12134493, and rs1320821 polymorphisms with migraine headache in a She population from the Fujian province in China. The alleles of rs4379368 were not in Hardy-Weinberg equilibrium in the migraine groups and the T allele of rs4379368 was more common than the C allele in subjects with migraine, possibly suggesting a selection bias for this allele in this population. In support of this, the CT and TT genotypes were more frequent in the migraine compared with the control groups (*P* = 0.019), while the presence of the CC genotype reduced the risk of migraine. These CT and TT genotypes were also more common in females with migraines than females without migraines. There was no difference in the frequency of the rs4379368 genotypes in males. These findings suggest that carrying the T allele of rs4379368 and being female puts you at risk for migraine headaches in this She population. Subgroup analysis indicated that subjects without aura had a lower frequency of the CC genotype than subjects with migraines with aura or healthy controls, suggesting genetic differencing in regard to migraine type. Univariate and multivariate analysis found that the AA and AG genotypes rs13208321 were associated with lower risk of migraine than the other alleles. However, none of the genotypes of this SNP were associated with migraine-related characteristics.

The alleles of rs105048561 were also not in Hardy-Weinberg equilibrium in either the migraine or control groups. The C allele was the most common allele in both groups. As this is the first study to describe the genetic properties of the rs105048561 loci, it is not known if this lack of Hardy-Weinberg equilibrium for rs10504856 is specific to the She population or is more general across other ethnic populations in China or other parts of the world. Additional studies are required to address this issue. For the other three alleles studied, there was no difference in allelic or genotypic frequency between the migraine and control groups.

A study by Chasman et al. [14] found that migraines can be subdivided not only be the presence or absence of an aura but also by a number of other features in including family history, frequency, duration, laterality, pulsing pain, nausea, photophobia, phonophobia, or aggreavation by physical activity. Other than the association of rs4379368 CC genotype with migraine with aura, none of the SNPs were associated with these other migraine-associated characteristics.

### Comparison of findings with prior studies

Our findings differ from the study of Anttila et al. [11] which found all five of the SNPs we investigated were associated with risk of migraine in a Western population. Our results also differ from those of Sintas et al. [12] that assessed the association of a subset of SNPs identified by Antilla et al. with migraine in a Spanish population. Sintas et al. evaluated 12 SNPs in a population of people who had migraine with aura (*n* = 512) and migraine-free controls (*n* = 535). They found a nominal association of four SNPs with migraine with aura: rs2651899 (within the PR domain containing 16 [*PRDM16*] gene), rs10166942 (near TRPM8), rs12134493 and rs10504861. In contrast to our findings, they saw no association of rs4379368 with migraine (*P* values >0.226). In addition, we found no association of rs1213449 or rs10504861 with migraine. Our study did not evaluate the association of rs2651899 or rs10166942 with migraine.

The difference in findings between our study and the two prior studies may result from ethnic/genetic differences between the study populations. Alternatively, the differences between the studies may reflect the small sample size of our study.

As mentioned previously, Anttila et al. found an association of rs4379368 and rs13208321with migraine [11]. Of note, the C allele of rs4379368 and the T allele of rs10504861 are the major alleles for these loci in Western countries [11], while in our population they are the minor alleles. Our study and that of Anttila et al. support the idea that rs4379368 and rs13208321 are genetic markers for migraine. The rs4379368 SNP is located within an intron in the transcript of the *C7orf10* gene, which encodes succinic HMG coenzyme A transferase [15]. The various alleles might differentially influence the transcription and/or RNA processing of the gene and consequently affect the risk of migraine. rs13208321 is located within the *FHL5* gene and may influence protein function.

The SNP rs12134493, located upstream of the *TSPAN2* gene, was not associated with migraine in our total study population but the CC genotype was more frequent in males with migraine compared with those without migraine. *TSPAN2* encodes a four transmembrane protein in cells which mediates signal transduction and is involved in the modulation of development, activation, growth, and movement of cells [16]. The role of the *TSPAN2* region of the genome in migraine is supported by the results of another genome-wide study by Esserlind et al. [17] that found another SNP (rs2078371) located near *TSPAN2* reached genome-wide significance for association with migraine in Danish and Icelandic populations. Our study and that of Esserlind et al. evaluated different sets of SNPs; Esserlind et al. assessed the top six SNPs identified in a prior genome-wide study migraine association study which did not include any of the SNPs we investigated [10]. The potential role of *TSPAN2* in migraine headaches is unclear. Larger multi-centered clinical studies with greater sample sizes in geographically and genetically diverse populations are required to elucidate the role of these SNPs in migraine headache susceptibility.

## Conclusion

Several large genome-wide association studies have identified genes that are possibly associated with the susceptibility of migraine headaches [10, 11, 18–21]. We investigated the relationship of five SNPs with susceptibility of migraine headaches in Chinese She subjects. Our findings suggest that carrying the T allele of rs4379368 and being female puts you at risk for migraine headaches in this She population. Our findings support the idea that rs4379368 and rs13208321 may be genetic marker for susceptibility for migraine headaches.

## Additional file

Additional file 1:
**Restriction pattern of different PCR products.** **Table S1.** Comparisons of migraine with or without aura, and controls.

## Abbreviations

SNP: Single nucleotide polymorphisms
*C7orf10*: Succinic HMG coenzyme A transferase gene
*MMP16*: Metallopeptidase 16 gene
*AJAP1*: Adherens junctions associated protein 1 gene
*TSPAN2*: Tetraspanin 2 gene
*FHL5*: Four and a half LIM domains protein 5 gene
PCR-RFLP: Polymerase chain reaction-restriction fragment length polymorphism
PCR: Polymerase chain reaction
bp: Base pairs
*PRDM16*: PR domain containing 16 gene
*TRMP8*: Transient receptor potential cation channel subfamily M member 8
