# Supplementary material for: Association of genetic loci for migraine susceptibility in the she people of China
Source: J Headache Pain. 2015 Aug 1;16:70. doi: 10.1186/s10194-015-0553-1 (PMC4522003; doi:10.1186/s10194-015-0553-1)
Supplement: Additional file 1: — Restriction pattern of different PCR products. Table S1. Comparisons of migraine with or without aura, and controls. [file 10194_2015_553_MOESM1_ESM.doc]

**Additional file 1**

Restriction pattern of different PCR products.

The following restriction patterns were identified:

- rs4379368: The length of the initial PCR products was 258 base pairs (bp). Following digestion with HphI, 3 genotypes could potentially be identified: CC (2 fragments: 179 bp and 79 bp), CT (3 fragments: 258 bp, 179 bp and 79 bp) and TT (1 fragment: 258 bp) (Supplementary Figure 1a).
- rs10504861: The intact PCR product was 166 bp. Following digestion with Tail, 3 genotypes were possible: CC (2 fragments: 103 bp and 63 bp), CT (3 fragments: 166 bp, 103 bp and 63 bp) and TT (1 fragment: 166 bp) (Supplementary Figure 1b).
- rs10915437: The full length PCR product was 410 bp. After MspA1I digestion 3 genotypes could potentially be identified: AA (2 fragments: 280 bp and 130 bp), AG (3 fragments: 410 bp, 280 bp and 130 bp) and TT (1 fragment: 410 bp) (Supplementary Figure 1c).

Supplementary Figure 1.


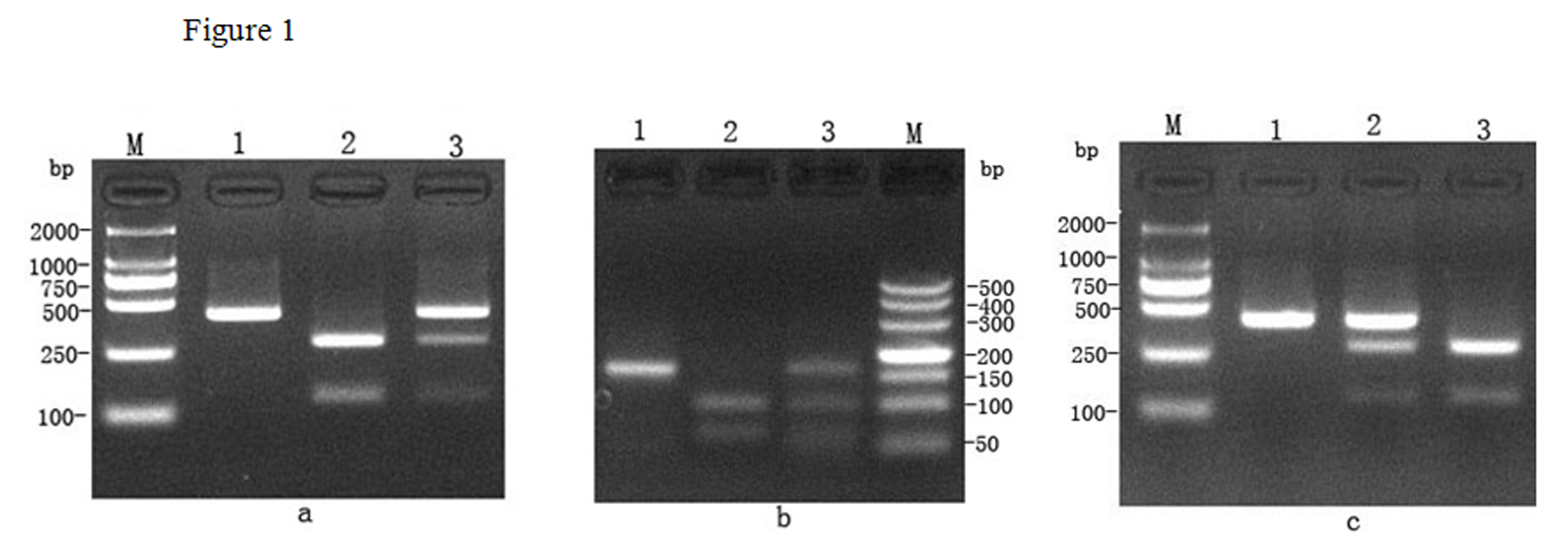


| **Table S1. Comparisons of migraine with or without aura, and controls** | | | | | | | | | | | | | |
| --- | --- | --- | --- | --- | --- | --- | --- | --- | --- | --- | --- | --- | --- |
|  | **rs4379368** | | |  | **rs10915437** | | |  | **rs12134493** | |  | **rs13208321** | |
| Clinical charateristics | CC | CT | TT |  | AA | AG | GG |  | AA or AC | CC |  | AA or AG | GG |
| Family history |  |  |  |  |  |  |  |  |  |  |  |  |  |
| No | 28 (12.4%) | 127 (56.4%) | 70 (31.1%) |  | 102 (45.3%) | 102 (45.3%) | 21 (9.3%) |  | 17 (7.6%) | 208 (92.4%) |  | 13 (5.8%) | 212 (94.2%) |
| Yes | 15 (20.0%) | 35 (46.7%) | 25 (33.3%) |  | 26 (34.7%) | 38 (50.7%) | 11 (14.7%) |  | 9 (12.0%) | 66 (88.0%) |  | 3 (4.0%) | 72 (96.0%) |
| Aura |  |  |  |  |  |  |  |  |  |  |  |  |  |
| No | 29 (12.2%) | 125 (52.5%) | 84 (35.3%)^b^ |  | 102 (42.9%) | 110 (46.2%) | 26 (10.9%) |  | 20 (8.4%) | 218 (91.6%) |  | 15 (6.3%) | 223 (93.7%) |
| Yes | 14 (22.6%) | 37 (59.7%) | 11 (17.7%) |  | 26 (41.9%) | 30 (48.4%) | 6 (9.7%) |  | 6 (9.7%) | 56 (90.3%) |  | 1 (1.6%) | 61 (98.4%) |
| Frequency |  |  |  |  |  |  |  |  |  |  |  |  |  |
| <1 time/month | 15 (14.4%) | 61 (58.7%) | 28 (26.9%) |  | 46 (44.2%) | 51 (49.0%) | 7 (6.7%) |  | 10 (9.6%) | 94 (90.4%) |  | 7 (6.7%) | 97 (93.3%) |
| 1-3 times/month | 20 (13.2%) | 80 (53.0%) | 51 (33.8%) |  | 69 (45.7%) | 62 (41.1%) | 20 (13.2%) |  | 13 (8.6%) | 138 (91.4%) |  | 8 (5.3%) | 143 (94.7%) |
| >=4 times/month | 8 (17.8%) | 21 (46.7%) | 16 (35.6%) |  | 13 (28.9%) | 27 (60.0%) | 5 (11.1%) |  | 3 (6.7%) | 42 (93.3%) |  | 1 (2.2%) | 44 (97.8%) |
| Duration, years^a^ | 8.7 (6.1) | 8.1 (7.2) | 8.0 (6.5) |  | 8.4 (7.1) | 8.2 (6.7) | 7.1 (6.6) |  | 8.3 (5.9) | 8.2 (6.9) |  | 11.0 (6.5) | 8.0 (6.8) |
| Laterality |  |  |  |  |  |  |  |  |  |  |  |  |  |
| Unilateral | 18 (17.8%) | 57 (56.4%) | 26 (25.7%) |  | 47 (46.5%) | 45 (44.6%) | 9 (8.9%) |  | 9 (8.9%) | 92 (91.1%) |  | 6 (5.9%) | 95 (94.1%) |
| Bilateral | 24 (12.1%) | 105 (53.0%) | 69 (34.8%) |  | 81 (40.9%) | 94 (47.5%) | 23 (11.6%) |  | 17 (8.6%) | 181 (91.4%) |  | 10 (5.1%) | 188 (94.9%) |
| Pulsing pain |  |  |  |  |  |  |  |  |  |  |  |  |  |
| No | 12 (12.6%) | 54 (56.8%) | 29 (30.5%) |  | 40 (42.1%) | 43 (45.3%) | 12 (12.6%) |  | 8 (8.4%) | 87 (91.6%) |  | 2 (2.1%) | 93 (97.9%) |
| Yes | 31 (15.1%) | 108 (52.7%) | 66 (32.2%) |  | 88 (42.9%) | 97 (47.3%) | 20 (9.8%) |  | 18 (8.8%) | 187 (91.2%) |  | 14 (6.8%) | 191 (93.2%) |
| Nausea |  |  |  |  |  |  |  |  |  |  |  |  |  |
| No | 26 (19.4%) | 71 (53.0%) | 37 (27.6%) |  | 52 (38.8%) | 65 (48.5%) | 17 (12.7%) |  | 14 (10.4%) | 120 (89.6%) |  | 6 (4.5%) | 128 (95.5%) |
| Yes | 17 (10.2%) | 91 (54.8%) | 58 (34.9%) |  | 76 (45.8%) | 75 (45.2%) | 15 (9.0%) |  | 12 (7.2%) | 154 (92.8%) |  | 10 (6.0%) | 156 (94.0%) |
| Photophobia/phonophobia | |  |  |  |  |  |  |  |  |  |  |  |  |
| No | 10 (9.5%) | 58 (55.2%) | 37 (35.2%) |  | 52 (49.5%) | 47 (44.8%) | 6 (5.7%) |  | 9 (8.6%) | 96 (91.4%) |  | 7 (6.7%) | 98 (93.3%) |
| Yes | 33 (16.9%) | 104 (53.3%) | 58 (29.7%) |  | 76 (39.0%) | 93 (47.7%) | 26 (13.3%) |  | 17 (8.7%) | 178 (91.3%) |  | 9 (4.6%) | 186 (95.4%) |
| Aggravation by physical activity | |  |  |  |  |  |  |  |  |  |  |  |  |
| No | 4 (16.7%) | 13 (54.2%) | 7 (29.2%) |  | 8 (33.3%) | 15 (62.5%) | 1 (4.2%) |  | 1 (4.2%) | 23 (95.8%) |  | 2 (8.3%) | 22 (91.7%) |
| Yes | 39 (14.1%) | 149 (54.0%) | 88 (31.9%) |  | 120 (43.5%) | 125 (45.3%) | 31 (11.2%) |  | 25 (9.1%) | 251 (90.9%) |  | 14 (5.1%) | 262 (94.9%) |

^a^Data were presented by mean and standard deviation.

^b^P<0.05.
